# Hepatitis B Virus-Specific Cellular Immunity Contributes to the Outcome of Occult Hepatitis B Virus Infection

**DOI:** 10.3389/fmicb.2022.850665

**Published:** 2022-04-07

**Authors:** Weiyun Zhang, Shengxue Luo, Tingting Li, Min Wang, Jieting Huang, Qiao Liao, Bochao Liu, Xia Rong, Linhai Li, Jean-Pierre Allain, Yongshui Fu, Chengyao Li

**Affiliations:** ^1^Department of Transfusion Medicine, School of Laboratory Medicine and Biotechnology, Southern Medical University, Guangzhou, China; ^2^Department of Laboratory Medicine, General Hospital of Southern Theater Command of PLA, Guangzhou, China; ^3^Department of Pediatrics, Shenzhen Hospital of Southern Medical University, Shenzhen, China; ^4^Guangzhou Blood Center, Guangzhou, China; ^5^Emeritus Professor, Department of Blood, University of Cambridge, Cambridge, United Kingdom

**Keywords:** HBV, core/pol peptides, cellular immune response, immune cytokines, HBV infection outcomes, blood donor population

## Abstract

There is little known of immunologic factors leading to the occurrence of occult HBV infection (OBI). Specific cellular immune response to hepatitis B virus (HBV) core/pol peptides was compared between blood donor populations, including 37 OBIs, 53 chronic HBV infections (CHB), 47 resolved infections, and 56 non-infected controls, respectively. The rate of CD4^+^/CD8^+^ T cell proliferation in OBI or CHB carriers was higher than in HBV resolved and non-infected individuals (*P* < 0.05). The intensity of IFN-γ-secretion T-cell response of OBI carriers was highest, followed by CHB and resolved infections, and non-infected individuals (*P* < 0.05). The frequency of intracellular IFN-γ and IL-17A CD4^+^/CD8^+^ and IL-21 CD4^+^ T-cell responses was significantly higher in resolved infections than in OBI or CHB carriers (*P* < 0.05), while the level of extracellular IL-17A of peripheral blood mononuclear cells (PBMCs) was higher in OBI and CHB carriers than in resolved infections (*P* < 0.01). The frequency of intracellular IL-10 CD4^+^ T-cell response in CHB, OBI, and resolved infections was higher than in HBV non-infected individuals (*P* < 0.01). Intracellular IL-10 CD8^+^ T cell and extracellular IL-10 T-cell responses were higher in CHB than in OBI (*P* = 0.012) or HBV resolved infections (*P* < 0.01). In conclusion, the higher level of effective T-cell response with IFN-γ, IL-17A, and IL-21 contributes to resolved infection outcome, while higher levels of suppressive T-cell response with IL-10 result in HBV chronicity. OBI is an intermediary status between HBV resolved and chronic infections, in which IL-21 effector and IL-10 suppressor T-cell responses play an important role in directing the outcome of HBV infection.

## Introduction

Hepatitis B virus (HBV) is a member of the hepadnaviridae that causes acute self-limited, subclinical, and acute or chronic infections, of which chronic hepatitis can lead to liver failure, cirrhosis, and hepatocellular carcinoma (HCC). An estimated 296 million people worldwide were living with chronic hepatitis B infection in 2019, with 1.5 million new infections each year, and 820,000 deaths were reported in 2019 from HBV-related liver diseases according to the WHO data sheet for Hepatitis B. Nearly half of them were in China (Zhang et al., 2017). The course of chronic HBV infection might be attributed to the interaction between virus replication in the liver and immune response in the host (Meng et al., 2019). HBV persistence was likely based on a weak immune response to the viral antigens (Rehermann and Nascimbeni, 2005; Peeridogaheh et al., 2018). HBV-induced cellular immune responses were manifested in T-cell proliferation and response, of which the strong response successfully could clear the virus in acutely infected patients, while the relatively weak response might relate with viral persistence in chronically infected patients (Liaw and Chu, 2009; Peeridogaheh et al., 2018). Meanwhile, specific T-cell response associated with the pathogenesis of HBV persistence and HBV replication was the key driver of immune-mediated liver injury and disease progression (Peeridogaheh et al., 2018).

Occult HBV infection (OBI) is characterized by the presence of very low levels of HBV DNA in circulation or liver tissue from individuals who are negative for hepatitis B surface antigen (HBsAg) (Raimondo et al., 2008, 2019). Mostly OBI carriers are antibody positive to hepatitis B core antigen (anti-HBc). There is a large amount of evidence showing the clinical relevance of OBI as a risk factor for liver cirrhosis and HCC, and HBV reactivation or transmission through liver transplant or blood transfusion (Wong et al., 2011; Niu et al., 2014; Coppola et al., 2016; Candotti et al., 2019; Mak et al., 2020).

Previous studies demonstrated that the development of chronic HBV infection (CHB) is closely related to viral mutation, host immune status, and environmental characteristics (Pollicino et al., 2007; Candotti et al., 2012; Zhang et al., 2018a). Occurrence of OBI has been found associated with low-level antibody to HBsAg (anti-HBs) or viral mutations which influence viral replication and HBsAg detection (Candotti et al., 2012; Zheng et al., 2015; Zhang et al., 2018a). However, over 30% incident OBIs in repeat blood donors had high levels of anti-HBs (>300 IU/L) and virus strains were mostly wild-type (Zheng et al., 2015; Tang et al., 2018), which might be associated with the poorly defined cellular immunity of OBI carriers. In this study, we investigated the role of cellular immune response in the outcome of OBI, CHB, resolved HBV infection (RHB), and HBV non-infection identified in Chinese blood donors.

## Materials and Methods

### Study Subjects

Blood donors were routinely screened between June 2016 and September 2017 at Guangzhou Blood Center for HBsAg and antibodies to hepatitis C virus (HCV), human immunodeficiency virus (HIV), and treponema pallidum by two different enzyme immunoassays (EIAs), respectively. The blood donors were further tested for HBV, HCV, and HIV genomes by nucleic acid testing (NAT) [Procleix Ultrio, individual donation NAT, Grifols Diagnostic Solutions, San Diego, CA, United States; limit of detection (LOD) HBV DNA 10.4 IU/mL] (Tang et al., 2018). The HBsAg and OBI carriers were tested by follow-up for HBsAg, anti-HBs, HBeAg, anti-HBe, and anti-HBc with chemiluminescent microparticle immunoassay (CMIA) in the Abbott Architect i2000SR analyzer (Abbott Diagnostic, Chicago, IL, United States). HBsAg and anti-HBs values <0.05 IU/mL or 10 IU/L, respectively, are considered negative. HBeAg or anti-HBc S/CO value <1 is considered negative, while anti-HBe S/CO value >1 is negative. HBV DNA was extracted from 1 to 2.5 ml of plasma with a large volume High Pure Viral Nucleic Acid Extraction kit (Roche Diagnostics, Mannheim, Germany). Viral load was quantified by real-time PCR (qPCR) as previously described (LOD HBV DNA 5 IU/mL) (Zhang et al., 2018a). OBI carriers were identified by the follow-up detection of different viral genomic regions at a minimum interval of 6 months (Tang et al., 2018; Raimondo et al., 2019).

This study was approved by the Medical Ethics Committee of Guangzhou Blood Center and followed the ethical guidelines of the 1975 Declaration of Helsinki. All study subjects signed an informed consent.

### Biochemistry Test

The levels of biochemistry parameters in blood were measured by an automatic biochemistry analyzer (Cobas c 702, Roche Diagnostics, Mannheim, Germany), including alanine transaminase (ALT), aspartate transaminase (AST), total bilirubin (TBIL), direct bilirubin (DBIL), total bile acid (TBA), albumin (ALB), adenosine deaminase (ADA), cholinesterase (CHE), γ-glutamyl transpeptidase (γ-GT), and total protein (TP).

### Cells and Hepatitis B Virus Peptides

Peripheral blood mononuclear cells (PBMCs) were isolated from EDTA anti-coagulated blood within 4 h of collection by Ficoll-Hypaque density gradient centrifugation (LSM, MP Biomedicals, LLC, United States). Freshly isolated PBMCs were used for testing of T-cell responses to HBV.

Hepatitis B virus core and polymerase (pol) peptides (genotype: B2; GenBank accession number: ACO05370.1) were synthesized commercially, including 9 HLA-I and 8 HLA-II restrictive core epitope peptides and 15 HLA-I and 10 HLA-II restrictive pol epitope peptides (Supplementary Table 1) (Brinck-Jensen et al., 2015; Zhang et al., 2018b; Tan et al., 2019). These core or pol peptides were prepared in a pool with 5 μg/mL of each peptide.

Peripheral blood mononuclear cells were cultured in RPMI 1640 (GIBCO) supplemented with 10% fetal bovine serum (FBS), 1% HEPES, 100 U/mL penicillin, and 100 μg/mL streptomycin.

### T Cell Proliferating Assay

Freshly isolated PBMCs (1 × 10^6^ per well) were stained with 5,6-carboxyfluorescein diacetate succinimidyl ester (CFSE), and then seeded in 24-well round bottom culture plates incubated for 6 days with HBV core/pol peptides (5 μg/mL each). Cells were stained with anti-CD3, anti-CD4, and anti-CD8 fluorescent antibodies (BD Pharmingen), and then analyzed by flow cytometry (BD FACSCanto II, BD Biosciences).

### ELISpot Assay

Triplicates of 2 × 10^5^ PBMCs per well were inoculated in 96-well plates pre-coated with anti-IFN-γ monoclonal antibody (Dakewe Biotech Co., Ltd., China), and incubated for 20 h with the pooled HBV core/pol peptides (5 μg/mL each). ELISpot was performed according to the manufacturer’s protocol. The spots were counted using an ELISpot-reader (Bio-Sys GmbH, Karben, Germany) and results were given as the mean number of spot-forming cells (SFCs) per 1 × 10^6^ cells ± SD.

### Intracellular Cytokine Staining

Peripheral blood mononuclear cells (1 × 10^6^ per well) were stimulated by HBV core/pol peptides (5 μg/mL each), and phorbol myristate acetate/ionomycin (PMA; positive control) or buffer control (negative control). After 2 h of incubation, 1 μl Brefeldin A protein transport inhibitor (BD GolgiPlug) was added and continued for incubation with an additional 12 h. At completion of incubation, cells were stained with anti-CD4, anti-CD8, and anti-CD3 antibodies (BD Pharmingen), then fixed and permeabilized. Intracellular cytokines were stained separately for interferon-γ (IFN-γ), tumor necrosis factor α (TNF-α), (interleukin-2) IL-2, IL-17A, IL-21, IL-10, and tumor growth factor-β (TGF-β) (BD Pharmingen). Last, cells were washed and analyzed by flow cytometry.

### Extracellular Cytokine Measurement

Peripheral blood mononuclear cells (1 × 10^6^ per well) were stimulated with HBV core/pol peptides, positive and negative controls as above. After 5 days of incubation at 37°C in 5% CO_2_, the cytokines IFN-γ, TNF-α, IL-2, IL-17A, IL-21, IL-10, and TGF-β in the supernatants of cell cultures were quantified by a cytometric bead array (CBA, Becton-Dickinson). Data were analyzed with CBA software, and cytokine concentration was determined by comparison with standard curves.

### Statistical Analysis

Data were presented as the mean ± SD or the median within a range. The normal distribution of measurement data was used for two independent sample *t*-tests; multiple comparisons between groups were analyzed by the one-way ANOVA test; the set comparison between groups in non-parametric data was analyzed by the Mann–Whitney *U* test. Statistically significant differences are indicated with asterisks (*P < 0.05, ^**^P < 0.01, and ^***^P < 0.001). All graphs are generated with GraphPad Prism 7 software.

## Results

### Classification of Subjects and Cohorts

A total of 193 blood donors were successfully recruited for participating in the follow-up detection of specific cellular immune response to HBV antigens. Among these participants, 37 donors HBsAg-/HBV DNA+/anti-HBc+ were classified as OBI carriers, 53 HBsAg+ as CHB carriers (1 with anti-HBs+), 47 anti-HBc+ and anti-HBs+ as spontaneously resolved HBV infection, 56 anti-HBs+ only (33 vaccinated) or HBV serological/genomic negative (23 no marker) as HBV non-infected individuals, respectively. None of the OBI or CHB carriers received any antiviral treatment for at least 6 months prior to blood donation. OBI carriers ranged in age between 19 and 55 years and all except one had normal ALT level. OBI carriers were all anti-HBc+ but HBeAg−, and 20 of them (54.1%) were anti-HBs+. Viral load quantified by qPCR ranged between <5 and 548 IU/mL, which was significantly lower than observed in the CHB group (median 81 vs. 8,600 IU/mL, *P* < 0.001). The clinical information and HBV infection markers from the four groups of blood donors are presented in Supplementary Table 2. There was no significant difference in gender between the groups, while age was significantly higher in OBI than in other groups (42.4 ± 10.8, *P* < 0.001). Liver function parameters level of TBIL and DBIL were higher in the OBI group than in other groups, but not statistically different (*P* = 0.203 and *P* = 0.294, respectively).

### Higher T-Cell Proliferation Rate in Occult HBV Infection and Chronic HBV Infection Carriers

**FIGURE 1:**
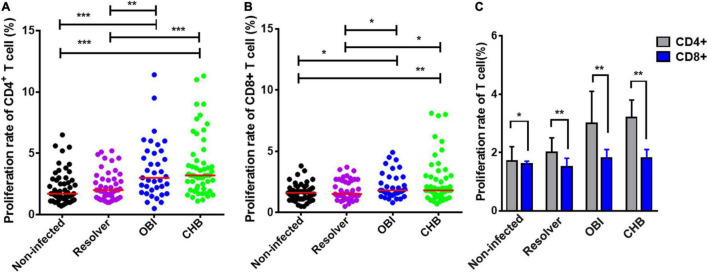
Proliferation rates of CD4^+^ and CD8^+^ T cells from blood donors with different outcomes of HBV infection stimulated with HBV core peptides. **(A,B)** HBV-core specific CD4^+^ or CD8^+^ T-cell proliferation rate. Each group median is indicated, and each dot represents a rate for T cell proliferation from four groups of individual blood donors. **(C)** Comparison of the median frequencies between CD4^+^ and CD8^+^ T cell proliferation rates in each study group. *P*-values are analyzed by one-way ANOVA and two-tailed *t*-test. Statistically significant differences are shown with asterisks (*P < 0.05, **P < 0.01, and ***P < 0.001).

The proliferation rates of CD4^+^ and CD8^+^ T lymphocytes stimulated with HBV core peptides were quantified by flow cytometry, which varied significantly between the four groups of blood donors with different HBV infection status (Figures 1A,B; *P* < 0.01 and *P* < 0.05). A specific CD4^+^ T cell proliferation rate was higher in OBI carriers (3.0%) and CHB carriers (3.2%) than in donors with resolved HBV infection (2%) or non-infected individuals (1.6%) (OBI vs. resolver or non-infection, *P* < 0.01; CHB vs. resolver or non-infection, *P* < 0.001; Figure 1A). Similar profiles were observed for specific CD8^+^ T cell proliferation rates between groups of blood donors, in which a significantly higher proliferation rate was observed in OBI and CHB carriers (both 1.8%) compared with resolved HBV infection (1.5%) and non-infected individuals (1.6%) (OBI vs. resolver or non-infection, *P* < 0.05; CHB vs. resolver or non-infection, *P* < 0.05; Figure 1B). The proliferation rates of CD4^+^ or CD8^+^ T cells between OBI and CHB carriers were not statistically different (*P* > 0.05). The median CD4^+^ and CD8^+^ T cell proliferation rates in each study group indicated that the proliferation rate of CD4^+^ T cells was significantly higher than that of CD8^+^ T cells in all four groups (*P* < 0.05; Figure 1C).

### Higher Interferon-γ Secreting T-Cell Response in Occult HBV Infection and Chronic HBV Infection Carriers

**FIGURE 2:**
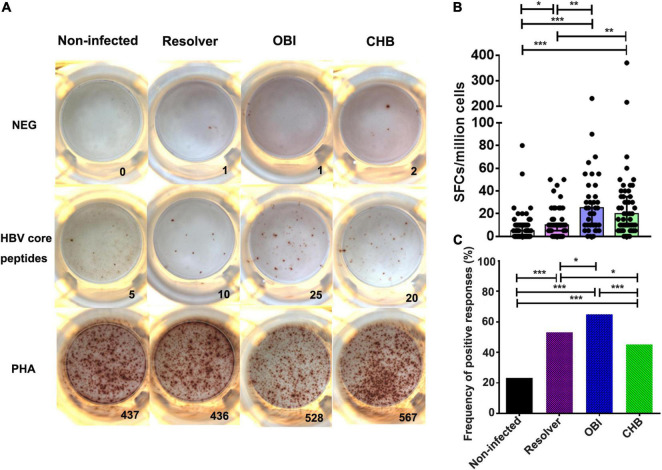
A specific IFN-γ secreting T-cell response of PBMCs to HBV core peptides between four groups of blood donors with different status of HBV infection by ELISpot. **(A)** Representative specific IFN-γ secreting T-cell response of PBMCs by ELISpot after stimulation with HBV core peptides. **(B)** The level of IFN-γ secreting T-cell response from individual donors is presented as a mean number of spot-forming cells per 1 × 10^6^ PBMCs in triplicates. Each dot represents a value over the cut-off from individual donors and the median is indicated for each group. **(C)** Comparison of the frequency of positive response obtained in the four groups. *P*-values are analyzed by one-way ANOVA and two-tailed *t*-test. Statistically significant differences are shown with asterisks (*P < 0.05, **P < 0.01, and ***P < 0.001).

A specific IFN-γ secretion T-cell response between groups of blood donors with various HBV infection outcomes was found significantly different by ELISpot after stimulation with HBV core peptides (Figure 2A). The number of specific IFN-γ secretion T cells was significantly higher in OBI carriers (25 SFC/10^6^ PBMCs), followed by CHB carriers (20 SFC/10^6^ PBMCs), than in HBV resolved infections (10 SFC/10^6^ PBMCs, *P* ≤ 0.004) or non-infected individuals (5 SFC/10^6^ PBMCs, *P* < 0.001), but not statistically different between OBI and CHB carriers (*P* > 0.05; Figure 2B). Individuals with resolved HBV infection had relatively higher specific IFN-γ-secreting T-cell response than HBV non-infected individuals (*P* = 0.03; Figure 2B).

The frequency of positive T-cell response for IFN-γ secretion was significantly higher in OBI carriers (64.9%) than in resolved HBV infection (53.2%), or in CHB carriers (45.3%) or non-infected individuals (23.2%), which was statistically different between groups (*P* < 0.05; Figure 2C).

### Higher Frequency of Intracellular Interferon-γ, Interleukin-17A, and Interleukin-21 Expressing T Cells in Resolved Hepatitis B Virus Infection Than in Occult HBV Infection and Chronic HBV Infection Carriers

**FIGURE 3:**
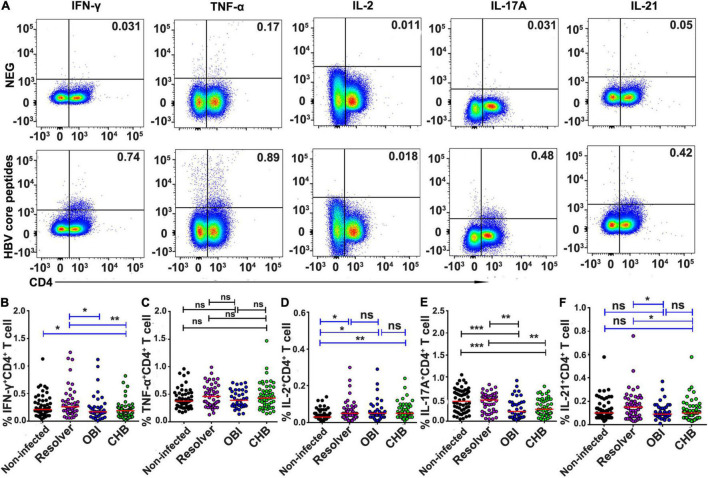
Frequency of intracellular cytokine expressing CD4^+^ T cells after stimulation with HBV core peptides. PBMCs were freshly isolated from individual blood donors and measured by ICS for intracellular IFN-γ, TNF-α, IL-2, IL-17A, or IL-21 secreting CD4^+^ T-cell response, respectively, after simulation with core peptides for 6 h. **(A)** Representative flow-cytometric analysis of reactivity of CD4^+^ T cells from an OBI donor stimulated with negative control and HBV core peptides. **(B–F)** The frequency of various intracellular cytokine secreting CD4^+^ T-cell response is expressed as a mean of triplicates from each individual. A median of frequency in each group is indicated and the differences between groups are compared with the Mann–Whitney test. Statistically significant differences are shown with asterisks (*P < 0.05, **P < 0.01, and ***P < 0.001); ns, no significant difference.

**FIGURE 4:**
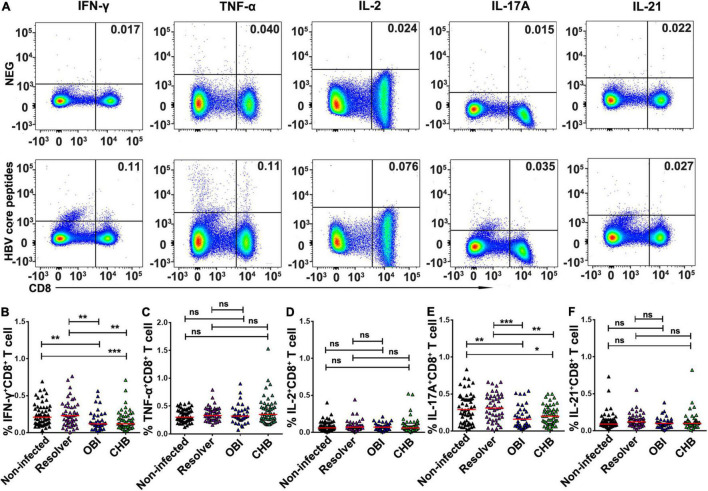
Frequency of intracellular cytokine expressing CD8^+^ T cells after stimulation of HBV core peptides. PBMCs were freshly isolated from individual blood donors and tested by ICS for intracellular IFN-γ, TNF-α, IL-2, IL-17A, or IL-21 secreting CD8^+^ T-cell response, respectively, after simulation with core peptides for 6 h. **(A)** Representative flow-cytometric analysis of reactivity of CD8^+^ T cells from an OBI donor stimulated with negative control and HBV core peptides. **(B–F)** The frequency of intracellular cytokine secreting CD8^+^ T-cell response was expressed as a mean of triplicates for each individual donor. A median of frequency in each group is indicated and the differences between groups are compared with the Mann–Whitney test. Statistically significant differences are shown with asterisks (*P < 0.05, **P < 0.01, and ***P < 0.001); ns, no significant difference.

After stimulation with HBV core peptides, the intracellular cytokines expressing CD4^+^ effective T-cell responses were individually quantified (Figure 3A). The frequency of intracellular IFN-γ (0.26%), IL-17A (0.48%), and IL-21 (0.15%) expressing CD4^+^ T cells was significantly higher in resolved HBV infection than in OBI (0.17, 0.22, and 0.09%) or CHB carriage (0.19, 0.27, and 0.10%), respectively (Figures 3B,E,F; *P* < 0.05). Any group of HBV infected blood donors had higher intracellular IL-2 expressing CD4^+^ T-cell response than HBV non-infected individuals (Figure 3D; *P* < 0.05), although there was no statistical difference in TNF-α^+^/CD4^+^ T-cell response between groups (Figure 3C; *P* > 0.05). Similarly, the frequency of intracellular cytokines expressing CD8^+^ effective T cells was tested (Figure 4A). The frequency of IFN-γ and IL-17A expressing CD8^+^ cells was significantly higher in resolved HBV infections (0.23 and 0.31%) or non-infected individuals (0.21 and 0.29%) than in those with OBI (0.12 and 0.16%) or CHB carriers (0.12 and 0.20%) (Figures 4B,E; *P* < 0.05), while the frequency of TNF-α, IL-2, and IL-21 expressing CD8^+^ T cells was not significantly different between groups (Figures 4C,D,F; *P* > 0.05).

### Higher Frequency of Intracellular Interleukin-10 and Tumor Growth Factor-β Expressing Suppressive T Cells in Chronic HBV Infection Carriers

**FIGURE 5:**
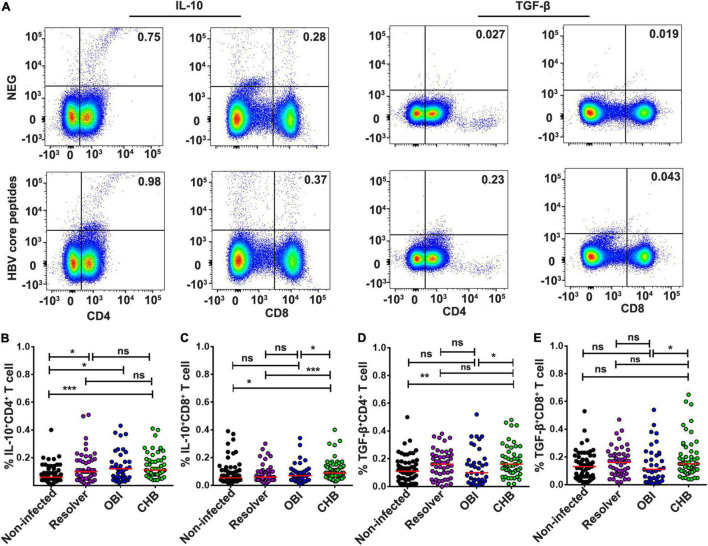
Intracellular IL-10 or TGF-β secretion CD4^+^/CD8^+^ suppressor T-cell responses to HBV core peptides. **(A)** Representative flow-cytometric analysis of reactivity of CD4^+^/CD8^+^ T cells from an OBI donor stimulated with negative control and HBV core peptides. **(B,C)** IL-10 secreting CD4^+^ or CD8^+^ T cells. **(D,E)** TGF-β secreting CD4^+^ or CD8^+^ T cells. A median of frequency in each group is indicated compared with the Mann–Whitney test. Statistically significant differences are shown with asterisks (*P < 0.05, **P < 0.01, and ***P < 0.001); ns, no significant difference.

The frequency of intracellular IL-10 expressing CD4^+^ suppressive T cells to HBV core peptides was higher in HBV infected individuals with OBI (0.12%), CHB (0.11%), or resolved infection (0.1%), respectively, than in HBV non-infected individuals (0.06%) (Figures 5A,B; *P* < 0.05). The frequency of intracellular IL-10 expressing CD8+ suppressive T cells was higher in CHB carriers (0.09%) than in HBV non-infected individuals (0.055%), resolved infections (0.06%), or OBI carriers (0.07%) (Figures 5A,C; *P* < 0.05). The frequency of intracellular TGF-β expressing CD4^+^ and CD8^+^ T cells was significantly higher in CHB carriers (both 0.16%) than in OBI carriers (0.10 and 0.11%) (Figures 5A,D,E; *P* < 0.05).

### Level of Extracellular Cytokines From Peripheral Blood Mononuclear Cells in Blood Donors With Different Hepatitis B Virus Infection Status

**FIGURE 6:**
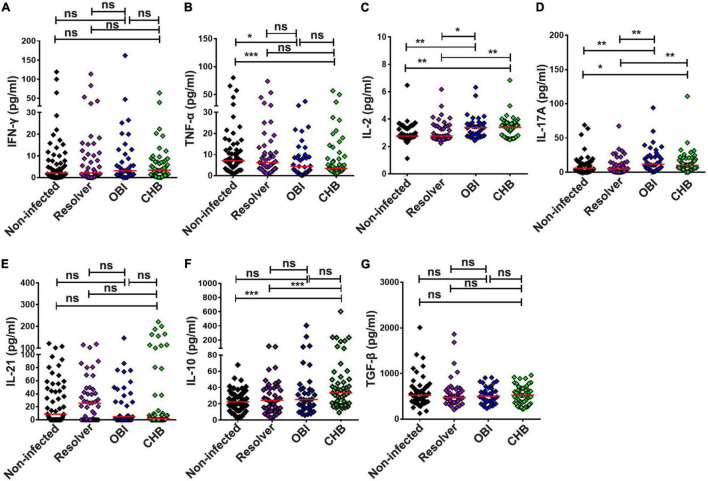
Extracellular cytokine concentrations in the culture supernatants of PBMCs stimulated by HBV core peptides. The concentration of IFN-γ **(A)**, TNF-α **(B)**, IL-2 **(C)**, IL-17A **(D)**, IL-21 **(E)**, IL-10 **(F)**, and TGF-β **(G)** in the supernatants of PBMCs was individually quantified by CBA after 72 h of stimulation. The result is expressed as the mean of triplicates. A median cytokine concentration in each group is indicated and the differences between groups are compared with the Mann–Whitney test. Statistically significant differences are shown with asterisks (*P < 0.05, **P < 0.01, and ***P < 0.001); ns, no significant difference.

Secretion of cytokines IFN-γ, TNF-α, IL-2, IL-17A, IL-21, IL-10, and TGF-β in culture supernatants of PBMCs was quantified after stimulation with HBV core peptides by cytometric bead array (Figure 6A). The level of TNF-α secreted by OBI (4.41 pg/mL) or CHB carriers (3.41 pg/mL) was lower than that obtained in HBV non-infected individuals (7.12 pg/mL) (Figure 6B; *P* < 0.05). The level of IL-2 was significantly higher in OBI (3.39 pg/mL) or CHB carrier cultures (3.40 pg/mL) than in resolved HBV infection (2.76 pg/mL) or non-infected individuals (2.76 pg/mL) (Figure 6C; *P* < 0.05). The level of IL-17A observed with OBI (11.82 pg/mL) or CHB carriers (10.12 pg/mL) was higher than with resolved HBV infection (4.57 pg/mL) or non-infected individuals (5.62 pg/mL) (Figure 6D; *P* < 0.05). The level of IL-21 varied insignificantly between the four groups of blood donors (Figure 6E; *P* > 0.05). The level of IL-10 in CHB carriers (33.62 pg/mL) was significantly higher than observed in resolved HBV infection (24.47 pg/mL) and non-infected individuals (22.4 pg/mL, *P* < 0.01), but was not statistically different from OBI carriers (25.2 pg/mL) (Figure 6F; *P* = 0.355). The level of secreting TGF-β between our four groups of blood donors was not statistically different (Figure 6G; *P* > 0.05).

### Cellular Immune Responses to Hepatitis B Virus Polymerase Peptides in Blood Donors With Different Hepatitis B Virus Infection Status

By using stimuli of HBV pol peptides, the specific cellular immune response was measured from the above four groups of blood donors with different HBV infection status (Supplementary Figures 1–5). The data showed that proliferation of CD4^+^ and CD8^+^ T cells was higher in OBI and CHB carriers (Supplementary Figure 1), IFN-γ secreting T-cell response was higher in CHB carriers (Supplementary Figure 2), intracellular IFN-γ, IL-17A, and IL-21 in CD4^+^ T-cell responses were lower (Supplementary Figure 3) but IL-10/CD8^+^ T-cell response (Supplementary Figure 4) and IL-10 secretion (Supplementary Figure 5) were higher in CHB carriers. These results were in a manner consistent with the cellular responses to HBV core peptides among these four groups of blood donors with various HBV infections.

## Discussion

Hepatitis B virus infections in adults are mostly self-limited, for whom the adaptive immune response and sustained T cell memory have been established for control of infection (Meng et al., 2019). HBV-specific T-cell response was efficiently induced in resolved HBV infection but deeply exhausted in CHB carriers. Previous studies found that cellular immune response played a significant role in the clearance of HBV (Suresh et al., 2019; Wang et al., 2020). Cellular response was involved in the process of hepatocyte inflammatory injury, and also assumed that low function was the main cause of viral persistence and the related chronic inflammation. HBV clearance and hepatic damage are mediated by the host immune response, which can suppress viral replication to extremely low levels and control the infection (Liaw and Chu, 2009; Karimzadeh et al., 2019).

Occult HBV infection can be infectious by blood transfusion and organ transplantation (Levicnik-Stezinar et al., 2008; Niu et al., 2014; Candotti et al., 2019). The natural history of OBI is partially understood. OBI may represent the final outcome of an unresolved infection with persistence of HBV DNA, which has not been cleared completely from the liver, or with partial or total lack of humoral immunity (Bläckberg and Kidd-Ljunggren, 2000; Raimondo et al., 2019). Solid evidence showed that OBI can coexist in a population of blood donors with high levels of anti-HBs (>1,000 IU/mL) (Raimondo et al., 2008; Wang et al., 2016; Tang et al., 2018), suggesting that the presence of neutralizing antibody could not be fully protective in some individuals.

Occult HBV infection appears as a mild infection. To shed further light on the natural history and pathogenesis of occult HBV infection, several lines of evidence beyond the response to HBsAg would support the spontaneous outcomes of HBV infected blood donors based on HBV specific T-cell response to HBcAg examined in this study.

First, four groups of blood donors carrying OBI (HBsAg-/HBV DNA+), CHB (HBsAg+/HBV DNA+), resolved HBV infection (anti-HBc+ only or plus anti-HBs+), and HBV non-infection (anti-HBs+ only or no HBV marker) were stratified by serological and genomic biomarkers of HBV infection. Besides viral factors, the specific cellular immune response to HBV core/pol peptides was compared among these four populations of blood donors.

Second, the level of effector and suppressor T-cell response to HBV core peptides differed significantly between OBI carriers, CHB carriers, resolved HBV infections, and non-infected individuals (*P* < 0.05), which is summarized in Supplementary Table 3. The HBV-specific CD4^+^/CD8^+^ T-cell proliferative responses were higher in OBI and CHB carriers than in resolved HBV infections or non-infected individuals (*P* < 0.05, Figures 1A,B). The frequency of specific IFN-γ secreting PBMCs was significantly higher in OBI carriers, followed by CHB carriers and HBV resolved infections compared with HBV non-infected individuals (*P* < 0.05, Figures 2B,C). The response of intracellular IFN-γ, IL-17A, and IL-21 expressing CD4^+^ or CD8^+^ effector T cells in HBV resolved infection was significantly higher than observed in OBI or CHB (*P* < 0.05, Figures 3B,E,F, 4B,E), while the concentration of circulating IL-17A was higher in OBI and CHB (*P* < 0.01, Figure 6D). In contrast, the level of intracellular or IL-10- secreting suppressor T-cell response was higher in CHB, followed by OBI, than in HBV infected resolvers or non-infected individuals (Figures 5B, 6F). The data suggest that CD4^+^/CD8^+^ T cells specifically expressing IFN-γ, IL-17A, and IL-21 play a role in resolving HBV infection, while the CD4^+^/CD8^+^ T cells expressing IL-10 played a suppressive role in the outcome of chronic HBV infection. However, OBI appeared closer to HBV resolved infection, which appears as a middle ground between resolved and chronic HBV infection.

Third, the function of HBV-specific cellular T-cell response has been partly explored for its role in the occurrence of occult HBV infection. By using ELISpot assay, a previous study described that blood donors with OBI or spontaneously resolved HBV infection produced a higher IFN-γ secreting T-cell response to recombinant HBcAg than those with inactive chronic HBV infection (HBeAg negative) or seronegative status (*P* < 0.05) (Bes et al., 2012). Using intracellular cytokine staining, a similar pattern of frequency of IFN-γ expressing CD4^+^/CD8^+^ T cells was observed in OBI, resolved HBV infection, and CHB carriers (Bläckberg and Kidd-Ljunggren, 2000). In our study, IFN-γ secreting T-cell response detected by ICS was lower in both OBI and CHB but higher when measured by ELISpot than in spontaneously resolved infection and non-infected individuals suggesting that IFN-γ secreting T-cell response played a significant role but might not be a critical factor contributing to the outcome of OBI or chronic HBV infection. A pattern similar to our observation indicated that HBsAg+ inactive carriers, OBI, and anti-HBc + only individuals had a relatively higher level of IFN-γ secreting CD4^+^ and CD8^+^ T-cell response to HBV core than HBV non-infected individuals (Shen et al., 2020). As IFN-γ secreting T cell did not significantly correlate with the outcome of HBV infection between resolved HBV infection, OBI and CHB carriage, the effective immune response relative to other specific cytokines associated CD4^+^ or CD8^+^ T cells might play a more crucial role in affecting the infection outcome. According to the data analysis here described (Supplementary Table 3), besides the controversial impact of intracellular or extracellular IL-17A expression CD4^+^/CD8^+^ T-cell response on the outcomes of HBV infection (Figures 3E, 4E, 6D), HBV specific IL-21 secreting CD4^+^ T-cell response appeared to play a significant role in spontaneous resolution of HBV infection (Figure 3F). The role of IL-21 associated T-cell response in viral clearance of HBV infection was clearly shown in previous studies (Li et al., 2013a,b, 2015b; Shen et al., 2017; Wang et al., 2018). Conversely to effective IL-21 immune response, IL-10 associated suppressive T-cell response had an important impact on HBV infection progressing toward chronicity (Das et al., 2012; Gong et al., 2015; Li et al., 2015a; Wang et al., 2018). In this study, intracellular IL-10 expressing CD4^+^ T-cell response was higher in HBV infected blood donors with CHB, OBI, or HBV resolution than in HBV non-infected individuals (Figure 5A), while intracellular IL-10 in CD8^+^ T cells and extracellular IL-10 response was higher in CHB carriers than seen in OBI carriers and resolved HBV infections (Figures 5B, 6F), suggesting that the higher level of IL-10 associated CD4^+^/CD8^+^ T-cell responses had a critical impact on progression to chronic HBV infection.

In summary, HBV-specific IFN-γ, IL-17A, and IL-21 secretion CD4^+^/CD8^+^ effector T-cell responses contributed to the resolution of viral infection, while IL-10 secretion CD4^+^/CD8^+^ T-cell responses contributed to progression to chronicity in the natural history of HBV infection, during which IL-21 and IL-10 T-cell responses played critical roles in the spontaneous resolution of HBV infection. OBI immune status was clearly intermediary between HBV resolved and chronic infection.

## Data Availability Statement

The original contributions presented in the study are included in the article/Supplementary Material, further inquiries can be directed to the corresponding author/s.

## Ethics Statement

The studies involving human participants were reviewed and approved by the Medical Ethics Committee of Guangzhou Blood Center. The patients/participants provided their written informed consent to participate in this study.

## Author Contributions

CL, YF, TL, WZ, LL, and SL designed the study. WZ, SL, MW, JH, QL, BL, and XR performed the experiments. WZ, SL, LL, J-PA, YF, and CL analyzed the data. LL, YF, and WZ provided the materials. WZ, SL, YF, J-PA, and CL wrote the manuscript. All authors contributed to the article and approved the submitted version.

## Conflict of Interest

The authors declare that the research was conducted in the absence of any commercial or financial relationships that could be construed as a potential conflict of interest.

## Publisher’s Note

All claims expressed in this article are solely those of the authors and do not necessarily represent those of their affiliated organizations, or those of the publisher, the editors and the reviewers. Any product that may be evaluated in this article, or claim that may be made by its manufacturer, is not guaranteed or endorsed by the publisher.
