# Supplementary material for: Hepatitis B Virus-Specific Cellular Immunity Contributes to the Outcome of Occult Hepatitis B Virus Infection
Source: Front Microbiol. 2022 Apr 7;13:850665. doi: 10.3389/fmicb.2022.850665 (PMC9022031; doi:10.3389/fmicb.2022.850665)
Supplement: Supplementary file 1 [file Data_Sheet_1.docx]

**Supplementary materials**

**Hepatitis B Virus-Specific Cellular Immunity Contributes to the Outcome of Occult Hepatitis B Virus Infection**

Weiyun Zhang^1,2*^, Shengxue Luo^1,3*^, Tingting Li^1^, Min Wang^4^, Jieting Huang^4^, Qiao Liao^4^, Bochao Liu^1^, Xia Rong^4^, Linhai Li^2^, Jean-Pierre Allain ^1,5^, Yongshui Fu^1,4^†, Chengyao Li^1^†

^1^ Department of Transfusion Medicine, School of Laboratory Medicine and Biotechnology, Southern Medical University, Guangzhou, China;

^2^ Department of Laboratory Medicine, General Hospital of Southern Theatre Command of PLA, Guangzhou, China;

^3^ Department of Pediatrics, Shenzhen Hospital of Southern Medical University, Shenzhen, China

^4^ Guangzhou Blood Center, Guangzhou, China;

^5^ Emeritus Professor, Department of Blood, University of Cambridge, Cambridge, UK.

* These authors contributed equally to this work.

**† Corresponding to:** Chengyao Li, Department of Transfusion Medicine, Southern Medical University, Guangzhou 510515, China (email: [chengyaoli@hotmail.com](mailto:chengyaoli@hotmail.com)); or Yongshui Fu, Guangzhou Blood Center, Guangzhou, China (email: fuyongshui1969@yahoo.com)

**Supplementary Table 1. HLA-I/II restricted HBV core and pol peptides**

| **HBV Core Peptides** | | **HBV Polymerase Peptides** | |
| --- | --- | --- | --- |
| HLA class Ⅰ restricted epitopes | | HLA class Ⅰ restricted epitopes | |
| Residues | Amino acid sequence | Residues | Amino acid sequence |
| core11-27 | ASVELLSFLPSDFFPSI | tp49-57 | NVSIPWTHK |
| core18-27 | FLPSDFFPSI | tp57-65 | KVGNFTGLY |
| core19-27 | LPSDFFPSI | tp63-71 | GLYSSTVPV |
| core88-96 | YVNVNMGLK | tp152-161 | TLWKAGILYK |
| core107-115 | CLTFGRETV | rt18-27 | TPARVTGGVF |
| core115-124 | VLEYLVSFGV | rt41-50 | LVVDFSQFSR |
| core117-125 | EYLVSFGVW | rt106-114 | GLSRYVARL |
| core139-148 | ILSTLPETTV | rt184-192 | SAICSVVRR |
| core141-151 | STLPETTVVRR | rt202-210 | YMDDVVLGA |
|  | | rt226-234 | FLLSLGIHL |
|  |  | rt306-314 | ALMPLYACI |
|  |  | rt318-327 | QAFTFSPTYK |
|  |  | rh63-71 | KYTSFPWLL |
|  |  | rh80-89 | ILRGTSFVYV |
|  |  | rh123-131 | SLYADSPSV |
| HLA class Ⅱ restricted epitopes | | HLA class Ⅱ restricted epitopes | |
| Residues | Amino acid sequence | Residues | Amino acid sequence |
| core1-20 | MDIDPYKEFGASVELLSFLP | tp98-112 | VGPLTVNENRRLKLI |
| core18-27 | FLPSDFFPSI | tp147-161 | RHYLHTLWKAGILYK |
| core28-47 | RDLLDTASALYREALESPEH | rt38-52 | ESRLVVDFSQFSRGN |
| core50-69 | PHHTALRQAILCWGELMNLA | rt65-79 | LQSLTNLLSSNLSWL |
| core111-125 | GRETVLEYLVSFGVW | rt73-87 | SSNLSWLSLDVSAAF |
| core117-131 | EYLVSFGVWIRTPPA | rt154-168 | LHLYSHPIILGFRKI |
| core120-139 | VSFGVWIRTPPAYRPPNAPI | rt269-283 | KQCFRKLPVNRPIDW |
| core147-156 | TVVRRRGRSP | rt317-331 | KQAFTFSPTYKAFLC |
|  |  | rh3-17 | LCQVFADATPTGWGL |
|  |  | rh76-90 | AANWILRGTSFVYVP |

**Supplementary Table 2. Characteristics of blood donors with various HBV infection status**

| Subjects | Non-infected | Resolved | OBI | CHB | *P* value |
| --- | --- | --- | --- | --- | --- |
| Numbers | 56 | 47 | 37 | 53 |  |
| Sex (male %) | 43 (76.8%) | 34 (72.3%) | 27 (73.0%) | 42 (79.2%) | *P*=0.834  *P*_1_=0.411  *P*_2_=0.266 |
| Age (year, mean±SD) | 31.9±9.2 | 38.2±10.0 | 42.4±10.8 | 33.0±8.8 | *P*<0.001  *P*_1_<0.001  *P*_2_<0.001 |
| ALT (U/L), median  range | 19.0  6-48 | 19.0  5-46 | 19.0  6-62 | 25.0  2-136 | *P*=0.051  *P*_1_=0.891  *P*_2_=0.044 |
| AST (U/L), median  range | 19.0  9-37 | 18.0  3-51 | 22.0  13-45 | 24.0  12-49 | *P*=0.000  *P*_1_=0.027  *P*_2_=0.131 |
| TBIL (μmol/L), median  range | 8.3  2.7-22.0 | 9.7  2.9-26.2 | 10.6  3.8-34.8 | 9.2  2.6-28.4 | *P*=0.203  *P*_1_=0.032  *P*_2_=0.130 |
| DBIL (μmol/L), median range | 3.7  1.5-8.1 | 3.9  1.6-9.6 | 4.2  1.9-9.5 | 3.6  1.4-8.8 | *P*=0.294  *P*_1_=0.042  *P*_2_=0.286 |
| TBA (μmol/L), median  range | 3.9  1.5-14.3 | 3.5  0.9-19.7 | 4.0  0.6-21.5 | 4.6  0.8-56.9 | *P*=0.561  *P*_1_=0.860  *P*_2_=0.315 |
| ALB (g/L), median  range | 45.0  37.1-52.9 | 45.9  37.8-54.1 | 46.7  42.0-51.4 | 48.0  41.4-55.4 | *P*<0.001  *P*_1_=0.004  *P*_2_=0.060 |
| ADA (U/L), median  range | 13.0  2-34 | 12.0  6-30 | 13.0  2-39 | 15.5  1-38 | *P*=0.842  *P*_1_=0.479  *P*_2_=0.625 |
| CHE (U/L), median  range | 8555  5246-13093 | 8707  4759-13440 | 8913  5203-11064 | 9148  5546-13888 | *P*=0.196  *P*_1_=0.997  *P*_2_=0.073 |
| γ-GT (U/L), median range | 23.0  8-118 | 24.0  5-59 | 25.0  9-128 | 24.5  10-116 | *P*=0.504  *P*_1_=0.522  *P*_2_=0.387 |
| TP (g/L), median  range | 71.9  59.9-81.2 | 72.9  59.6-80.8 | 73.5  66.2-79.9 | 74.9  63.6-82.2 | *P*=0.059  *P*_1_=0.272  *P*_2_=0.167 |
| HBcAb | - | + | + | + |  |
| HBsAb^+^/total (%) | 33/56  58.9% | 43/47  91.5% | 20/37  54.1% | 1/53  1.9% |  |
| HBsAb (IU/L), median range | 39.8  0.1->1000 | 175.3  3.7->1000 | 14.5  0.4-333.4 | 0.3  0-123.3 |  |
| HBeAg^+^/total (%) | 0 | 0 | 0 | 11/53  20.8% |  |
| HBeAb^+^/total (%) | 0 | 24/47  51.1% | 19/37  51.4% | 42/53  79.2% |  |
| HBV DNA (IU/ml), median  range | UD | UD | 81  <5-548 | 8.6E+03  7.6E+01-3.7E+09 |  |

*P*, comparison among four groups; *P*_1_, OBI vs. Non-infected; *P*_2_, OBI vs. CHB; UD, undetectable.

**Supplementary Table 3. Significant difference of specific T cell response to HBV core peptides between blood donors with various HBV infection status (median value)**

| Assays | Non-infected | Resolved | OBI | CHB |
| --- | --- | --- | --- | --- |
| Proliferation (%) |  |  |  |  |
| CD4^+^ | 1.6 | 2 | **3.0** | **3.2** |
| CD8^+^ | 1.6 | 1.5 | **1.8** | **1.8** |
| Elispot (SFC/10^6^ cells) | 5 | 10 | **25** | **20** |
| ICS (%) |  |  |  |  |
| IFN-γ CD4^+^ | 0.21 | **0.26** | 0.17 | 0.19 |
| IL-2 CD4^+^ | 0.03 | **0.05** | **0.05** | **0.05** |
| IL-17A CD4^+^ | 0.45 | **0.48** | 0.22 | 0.27 |
| IL-21 CD4^+^ | 0.1 | **0.15** | 0.09 | 0.1 |
| IL-10 CD4^+^ | 0.06 | 0.1 | 0.12 | **0.11** |
| TGF-β CD4^+^ |  |  | 0.10 | **0.16** |
| IFN-γ CD8^+^ | 0.21 | **0.23** | 0.12 | 0.12 |
| IL-17A CD8^+^ | 0.29 | **0.31** | 0.16 | 0.2 |
| IL-10 CD8^+^ | 0.055 | 0.06 | 0.07 | **0.09** |
| TGF-β CD8^+^ |  |  | 0.11 | **0.16** |
| CBA (pg/ml) |  |  |  |  |
| TNF-α | **7.12** | 6.19 | 4.41 | 3.41 |
| IL-2 | 2.76 | 2.76 | **3.39** | **3.4** |
| IL-17A | 5.62 | 4.57 | **11.82** | **10.12** |
| IL-10 | 22.4 | 24.47 | 25.2 | **33.62** |

The red number indicates that the median value of group is statistically higher than other groups in the category (*P*<0.05).


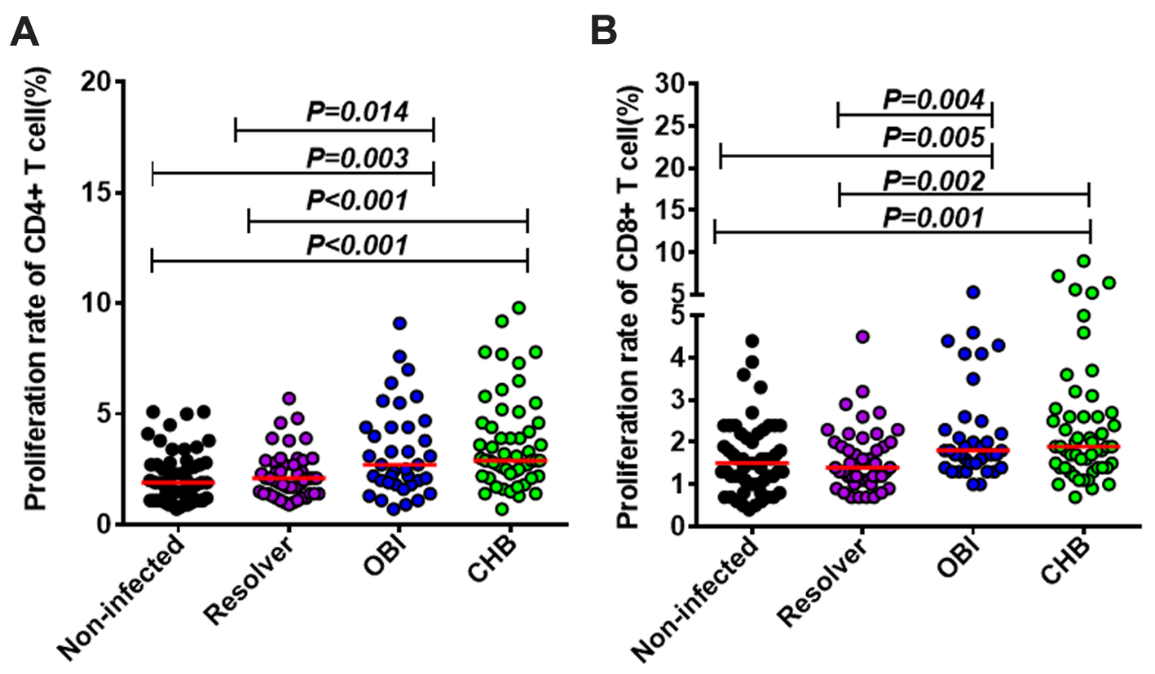


**Supplementary Figure 1. Proliferation of CD4^+^ and CD8^+^ T cells to HBV Polymerase peptides.**


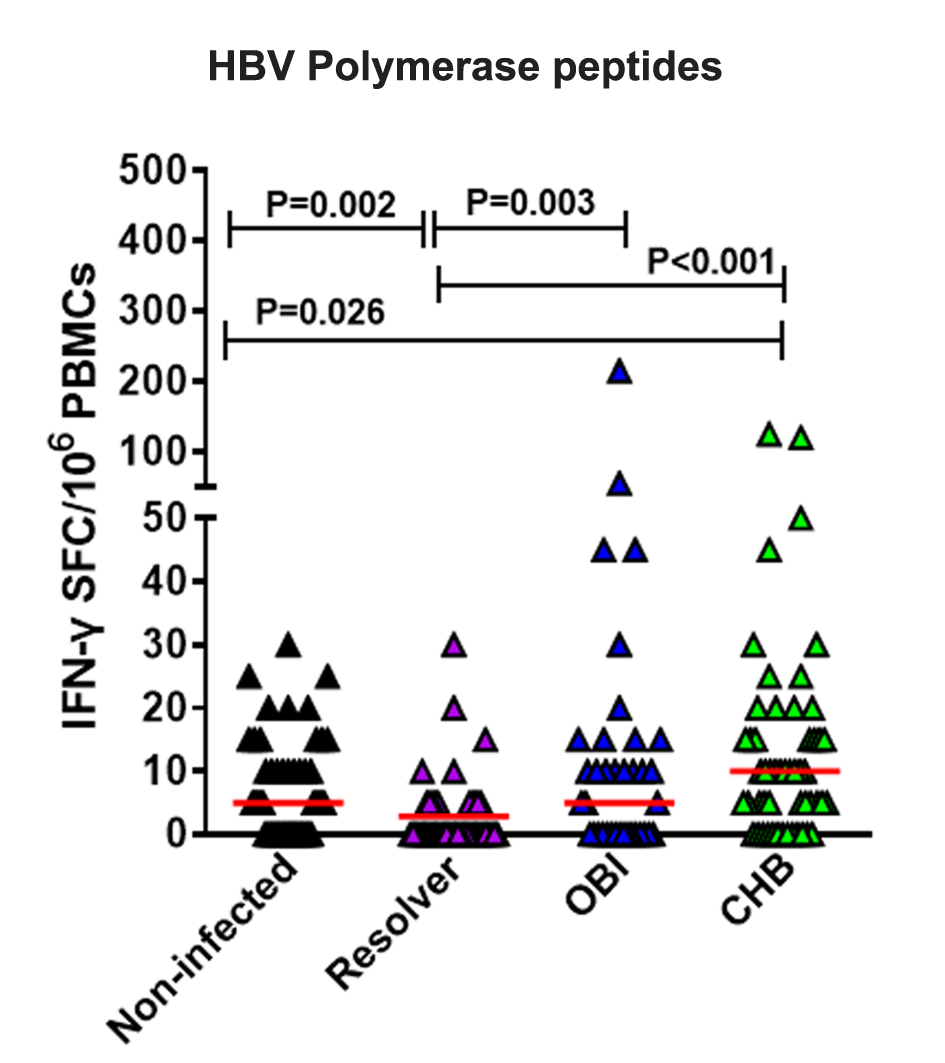


**Supplementary Figure 2. Specific IFN-γ secreting T cell response of PBMCs to HBV pol peptides.**


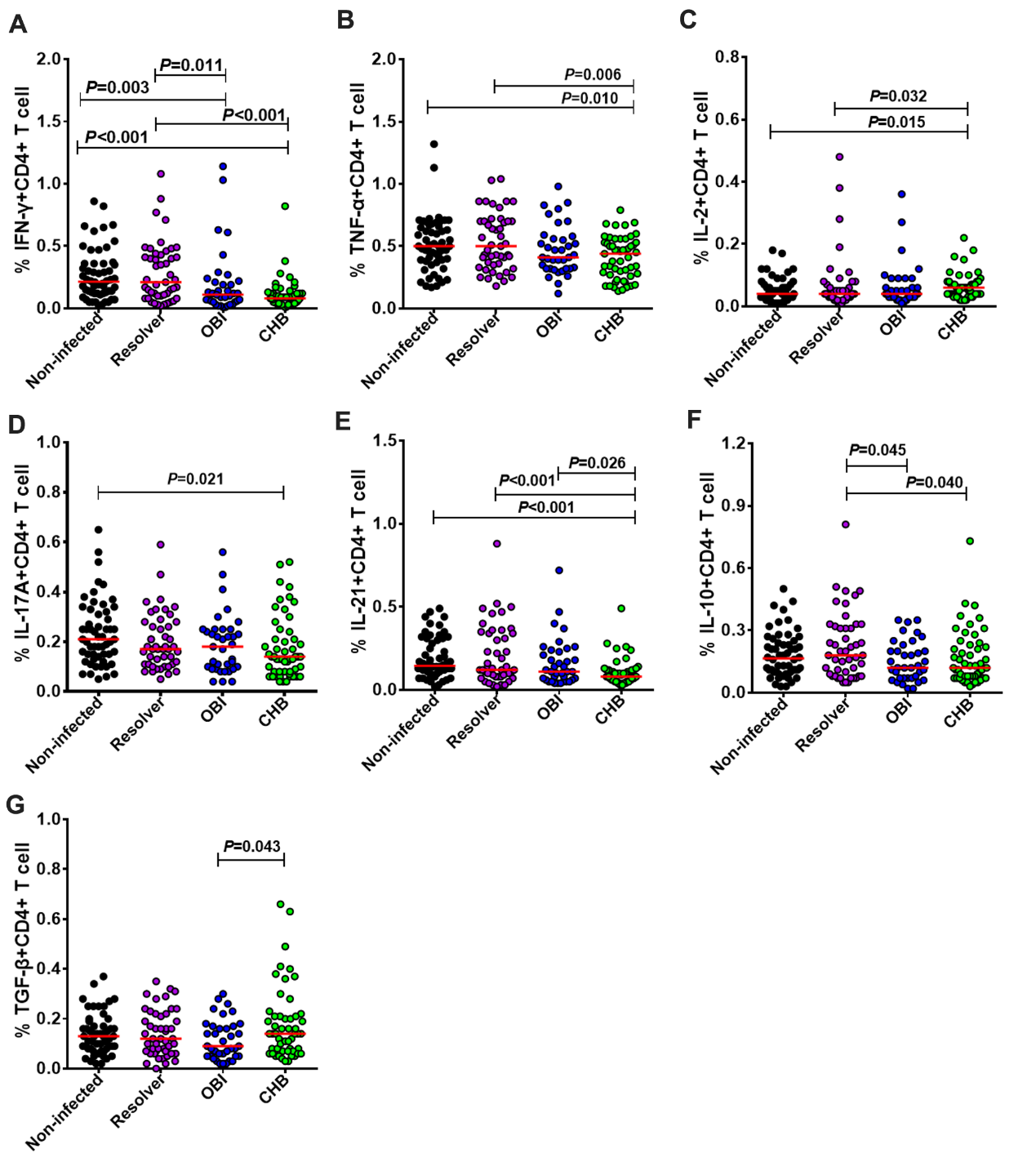


**Supplementary Figure 3. Intracellular cytokine expressing CD4^+^ T cell response to HBV Polymerase peptides.**


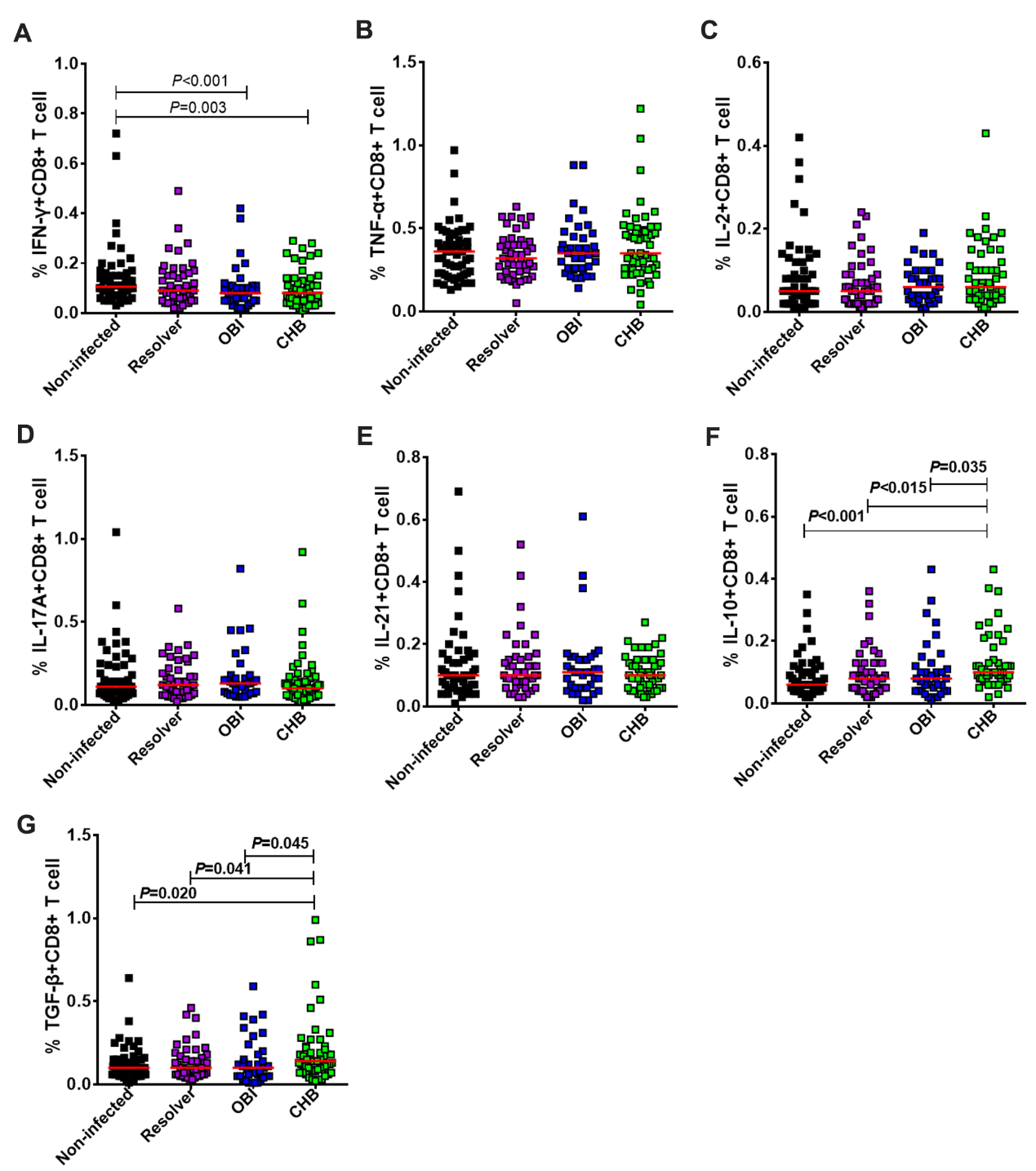


**Supplementary Figure 4. Intracellular cytokine expressing CD8^+^ T cell response to HBV Polymerase peptides.**


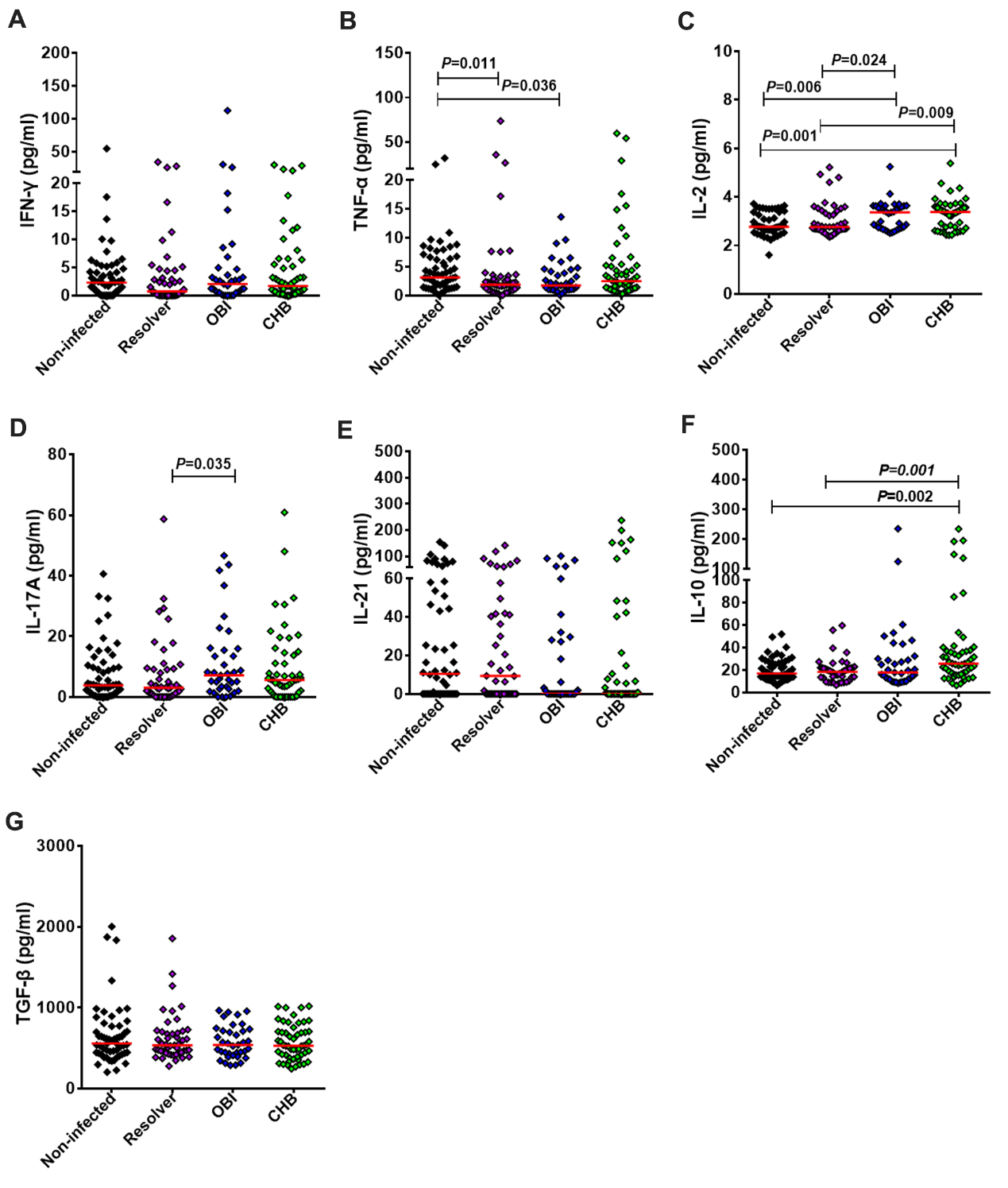


**Supplementary Figure 5. Secretion of cytokines in the culture supernatants of PBMCs to HBV Polymerase peptides.**
